# Giant Peripheral Osteoma of the Mandibular Antegonial Notch in an Elderly Patient: A Case Report

**DOI:** 10.7759/cureus.98781

**Published:** 2025-12-09

**Authors:** Yasuhisa Sawai, Eiji Mitate, Yota Yamauchi, Miho Hasumoto, Satoshi Wada, Hiroyuki Nakano

**Affiliations:** 1 Department of Oral and Maxillofacial Surgery, Kanazawa Medical University, Uchinada, JPN

**Keywords:** antegonial notch, compact osteoma, giant osteoma, mandible, peripheral

## Abstract

Peripheral osteoma is a benign bone tumor that frequently arises in the craniofacial skeleton. In the mandible, it usually involves the body or condyle, whereas lesions at or near the antegonial notch are extremely rare, particularly when they are large and occur in elderly patients. This report describes the case of an 80-year-old man who presented with an asymptomatic hard swelling in the left submandibular region. The maximal interincisal mouth opening was approximately 40 mm, which was within normal limits, and there was no mandibular deviation. Imaging revealed a pedunculated, homogeneously radiodense mass measuring 50 × 45 × 40 mm arising from the cortical surface of the left mandibular antegonial notch without cortical destruction or associated soft tissue mass. The lesion was removed en bloc via an extraoral submandibular approach together with a thin margin of normal cortical bone, and the markedly thinned mandibular cortex was reinforced with a titanium plate. Histopathological examination showed dense lamellar bone with small marrow spaces and no cellular atypia, consistent with compact-type peripheral osteoma. The postoperative course was uneventful apart from transient weakness of the marginal mandibular branch of the facial nerve, and no recurrence has been observed during 15 months of follow-up. In this case, the exact onset and growth rate of the lesion could not be determined because previous radiographs were unavailable, and relatively rapid enlargement over a short period could not be excluded. Chronic mechanical stimulation and local circulatory changes in the masseter region may have contributed to tumor development and growth; however, their roles remain speculative. This case highlights the importance of considering peripheral osteoma in the differential diagnosis of unilateral mandibular swelling, especially in atypical locations, and underscores the utility of detailed imaging evaluation and long-term postoperative surveillance.

## Introduction

Osteoma is a benign bone tumor characterized by the proliferation of mature bony tissue and occurs predominantly in the craniofacial skeleton [1]. Its growth is typically extremely slow, and the lesion may remain asymptomatic for a prolonged period, often being discovered only when facial deformity, malocclusion, or dysphagia becomes apparent [2].

Peripheral osteomas arise from the periosteum and form exophytic masses attached to the cortical surface. They are generally regarded as slowly enlarging lesions with a favorable prognosis [1-3]. In the mandible, peripheral osteomas most frequently involve the body and condylar region, whereas lesions arising near the angle or antegonial notch are exceptionally uncommon [4-6]. Only a few cases of peripheral osteoma at the mandibular antegonial notch have been documented in the literature, and some of these lesions reached a considerable size before being detected [5,7,8]. Because of their rarity and deep location, such tumors may escape notice until they cause visible facial asymmetry or functional disturbance.

Here, we report a rare case of a giant peripheral osteoma arising at the mandibular antegonial notch in an elderly patient and describe its clinical, radiological, and histopathological features. Possible etiologic factors, including mechanical stimulation and local circulatory changes, are also discussed.

## Case presentation

An 80-year-old man was referred to our department in January 2024 with a chief complaint of swelling in the left submandibular region. The patient’s medical history included diabetes mellitus and bilateral knee osteoarthritis. There was no notable family history. According to the patient and his referral documents, no swelling was noted in the left mandible when he underwent dental treatment at a local dental clinic two months prior to the current referral. It was during a maintenance visit to the same clinic in January 2024 that the swelling of the left mandible was noticed, and he was thus referred to our department for detailed examination and treatment.

**Figure 1:**
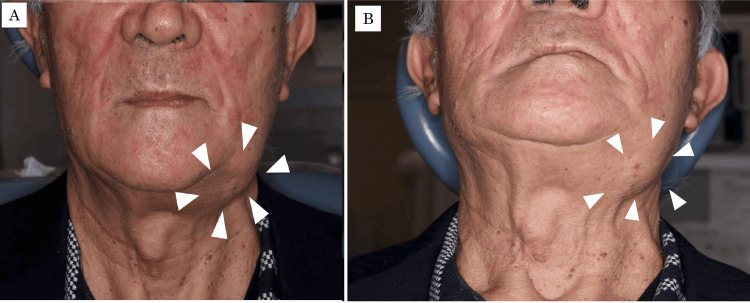
Extraoral photographs at first visit to our department A hard swelling (arrowhead) is evident in the left antegonial region of the mandible. (A) Frontal view; (B) inferior view.

On general examination, the patient’s height was 160 cm, and body weight was 59.2 kg; his nutritional status was good. Extraorally, his facial appearance was square-shaped, and a hard, non-tender swelling was observed in the left antegonial region of the mandible (Figure 1).

**Figure 2:**
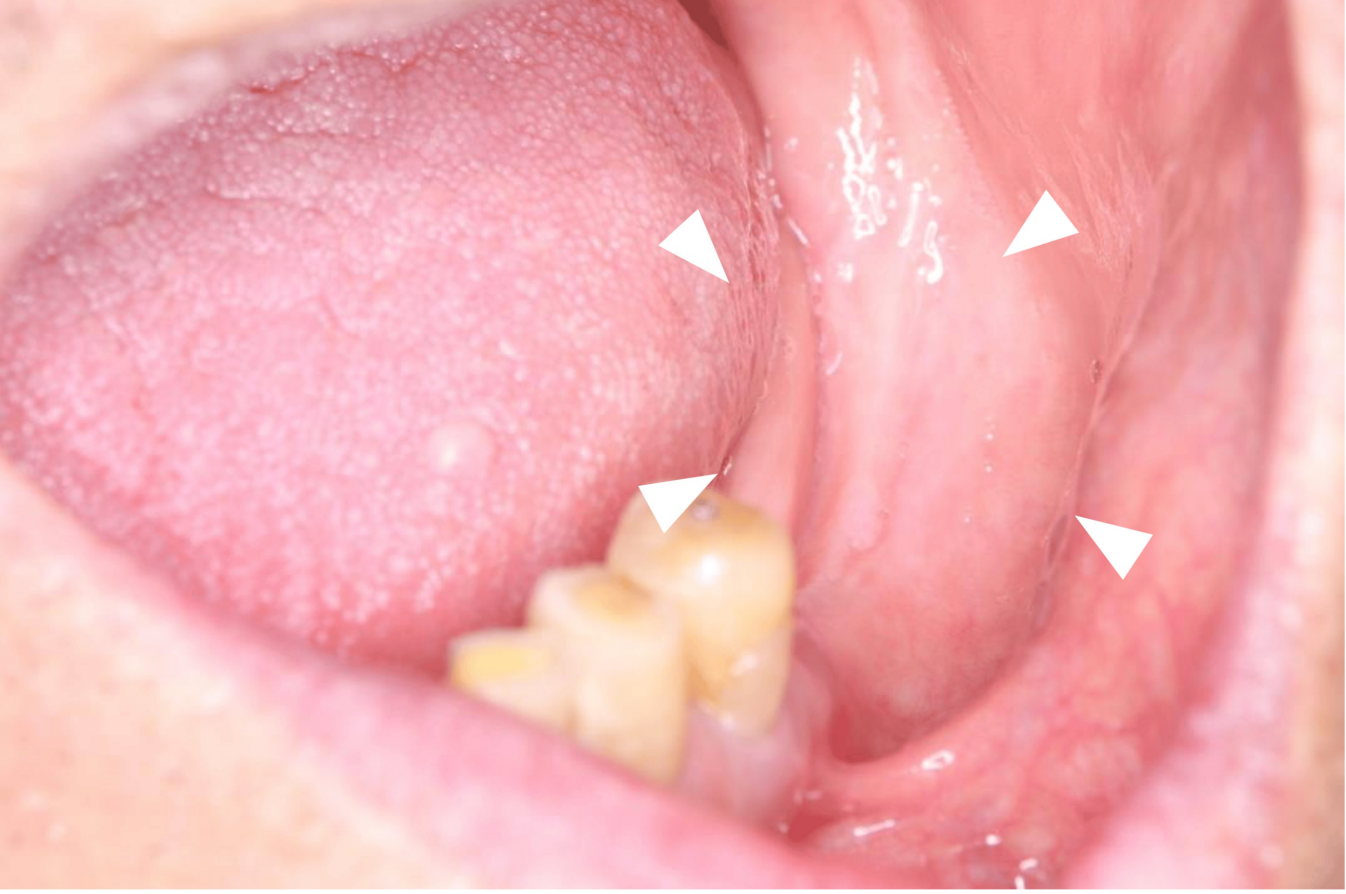
Intraoral photograph at first visit The gingiva in the left mandibular molar region appears normal in color. No swelling is observed in the vestibule or alveolar ridge.

The overlying skin showed no color change, ulceration, or adhesion to the mass. Intraorally, the gingiva in the left mandibular molar region appeared normal in color without buccal or alveolar enlargement (Figure 2).

**Figure 3:**
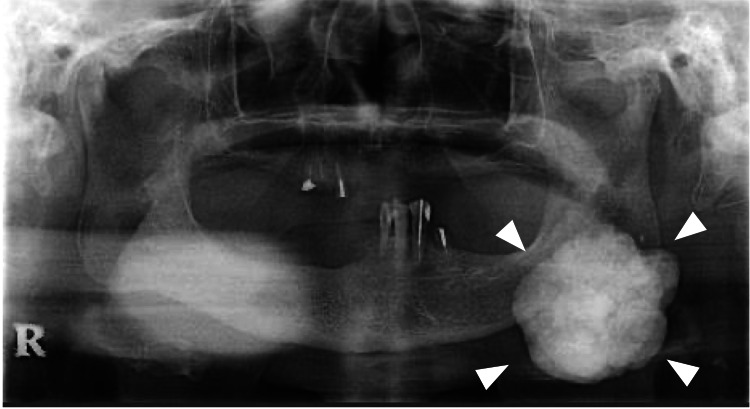
Panoramic radiograph at first visit A radiopaque mass with an irregular, lobulated border is seen at the anteroinferior border of the left mandibular angle (arrowhead).

The maximal interincisal mouth opening was approximately 40 mm, which was within normal limits, and there was no deviation of the mandible on opening or closing. Panoramic radiography revealed a multilobulated, irregularly contoured radiopaque mass arising from the anteroinferior border of the left mandibular angle (Figure 3). Severe residual ridge resorption was noted in both maxillary and mandibular molar regions.

**Figure 4:**
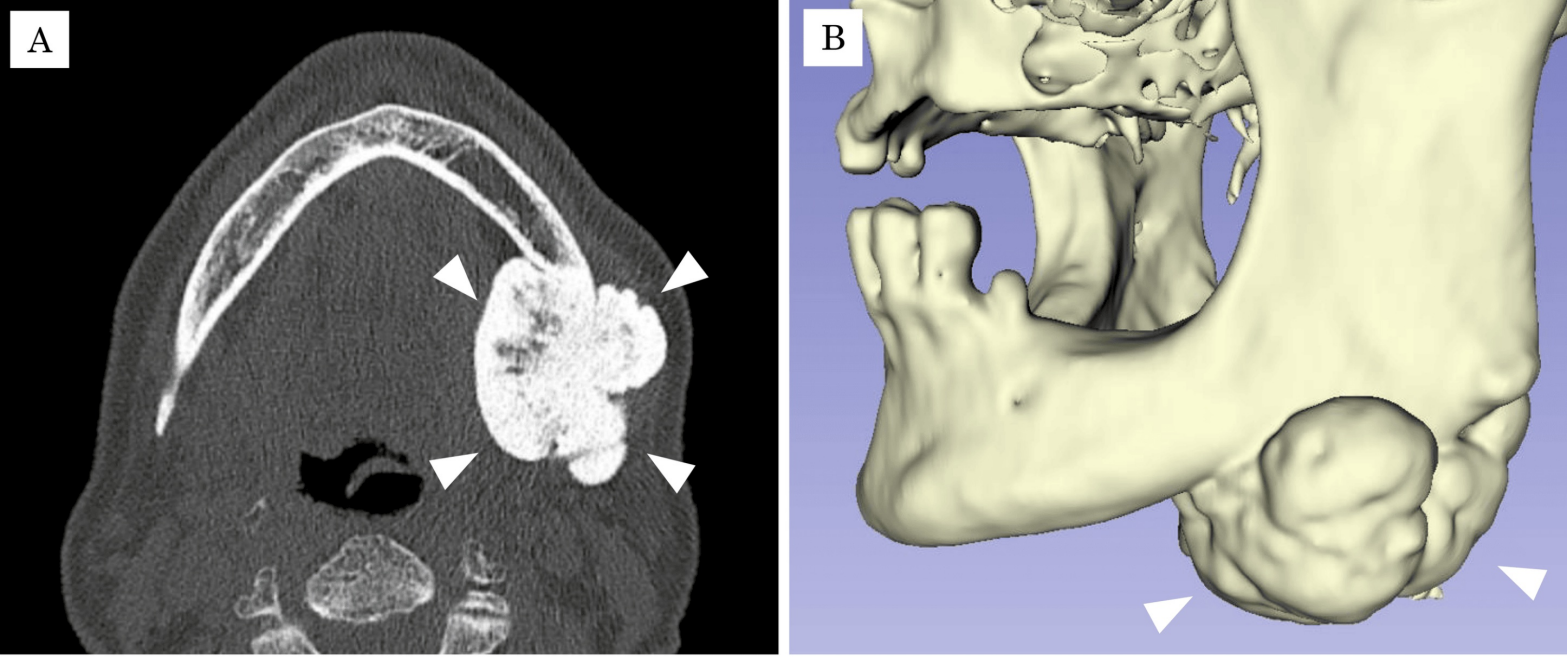
CT images at first visit (A) Axial view showing a pedunculated mass (50 × 42 × 40 mm) with an irregular surface extending over the buccal and lingual aspects of the left mandibular antegonial notch (arrowhead). (B) Three-dimensional reconstructed CT image.

Computed tomography (CT) and three-dimensional CT demonstrated a pedunculated mass measuring 50 × 42 × 40 mm extending over both the buccal and lingual aspects of the left mandibular antegonial notch (Figure 4).

The internal density was predominantly homogeneous and comparable to cortical bone, with small areas showing attenuation similar to cancellous bone. The lesion had a well-demarcated border and was attached to the mandibular cortex by a stalk. The mandibular canal was displaced slightly superiorly but remained intact. No surrounding soft tissue mass or cervical lymphadenopathy was observed.

Routine laboratory blood tests revealed no abnormalities. Based on the clinical and radiological findings, a provisional diagnosis of peripheral osteoma arising from the left mandibular antegonial notch was made.

**Figure 5:**
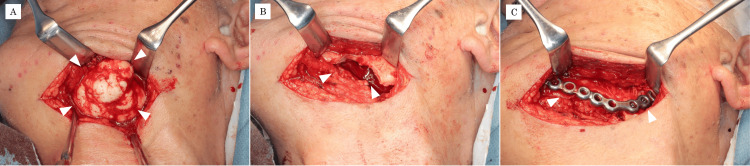
Intraoperative photographs (A) A submandibular skin incision is made approximately 3 cm below the inferior border of the mandible, and blunt dissection is performed to expose the lesion. (B) Surgical field after tumor resection. (C) Because of concern about fracture of the thinned mandibular cortex, a titanium plate is applied to reinforce the mandible.

Under general anesthesia, tumor resection was performed via an extraoral approach in February 2024. A 10 cm skin incision was made approximately 3 cm inferior to the lower border of the left mandible in the submandibular region, and blunt dissection was carried down to expose the tumor (Figure 5A).

The facial artery and vein were located superficial to the lesion and were ligated and divided. The submandibular gland was displaced by the mass. Identification and preservation of the marginal mandibular branch of the facial nerve were difficult because of the size and position of the tumor.

The lesion was resected en bloc from its base using a reciprocating saw and chisels (Figure 5B). To avoid damaging the tumor, part of the surrounding normal bone was included in the resection margin. Because the remaining mandibular cortex at the resection site was extremely thin and there was concern about postoperative fracture, the mandible was reinforced with a titanium plate (Figure 5C).

**Figure 6:**
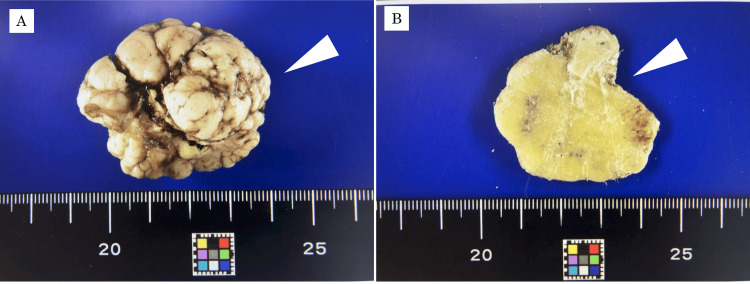
Resected specimen (A) Gross appearance showing a 50 × 45 × 40 mm whitish, multilobulated, hard mass with a smooth surface. (B) Cut surface showing that the interior of the lesion is solid and bone-hard.

**Figure 7:**
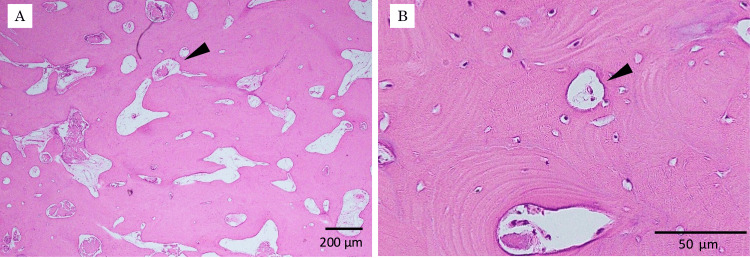
Histopathological findings (A) Hematoxylin and eosin stain (×4). Bone tissue containing marrow spaces is observed. (B) Hematoxylin and eosin stain (×40). Lamellar bone with focal marrow spaces containing blood vessels is observed (arrowhead). There is proliferation of relatively compact bone tissue with well-formed lamellar structures and marrow spaces, consistent with mature lamellar bone.

The resected specimen was a whitish, hard mass with an irregular, multilobulated surface, measuring 50 × 45 × 40 mm (Figure 6A). On the cut section, the lesion was solid and composed of dense bone (Figure 6B). After gross examination, the specimen was fixed in neutral buffered formalin, embedded in paraffin, sectioned, deparaffinized in xylene, dehydrated in ethanol, and stained with hematoxylin and eosin for histopathological evaluation. Histopathological examination revealed proliferation of mature bone tissue forming lamellar structures with marrow spaces. The bone was relatively compact, with well-organized lamellae and no cellular atypia (Figure 7).

**Figure 8:**
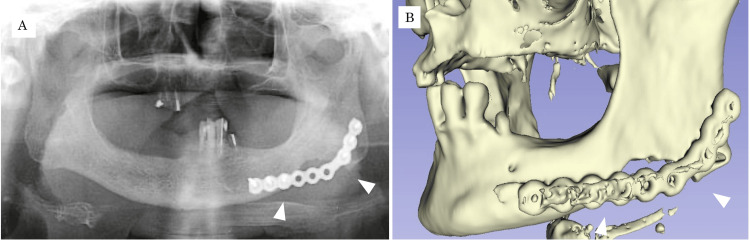
Imaging findings one week after surgery. (A) Panoramic radiograph; (B) three-dimensional CT image.

Based on these findings, a diagnosis of compact-type peripheral osteoma was established. Postoperatively, no neurosensory disturbance of the inferior alveolar nerve was observed. However, the patient exhibited reduced movement of the left oral commissure, suggestive of marginal mandibular nerve weakness. Oral mecobalamin at 1500 μg/day was prescribed, and the paralysis resolved within three months. Radiographs obtained one week after surgery showed no evidence of residual tumor (Figure 8), and no tumor recurrence has been observed during 15 months of follow-up.

## Discussion

Osteomas are benign tumors characterized by the proliferation of mature bone and are classified according to their site of origin into central osteomas arising within bone, peripheral osteomas arising from the periosteum, and extraskeletal (parosteal) osteomas occurring in soft tissues [1-3]. They occur most frequently in the craniofacial region, including the calvarium and paranasal sinuses, whereas involvement of the mandible is relatively uncommon [4,9,10]. When the mandible is affected, the most common sites are the inner and outer aspects of the mandibular angle, the inferior border of the chin, and the lingual aspect of the molar region [4,5].

Histopathologically, osteomas are categorized into compact osteoma, composed of dense lamellar bone with Haversian systems, and cancellous osteoma, which contains more trabecular bone with marrow spaces; compact osteomas are generally more common [1-3]. In the present case, the lesion consisted predominantly of dense lamellar bone with relatively small marrow spaces, consistent with a compact-type peripheral osteoma.

The etiology of osteoma remains incompletely understood, and various hypotheses have been proposed. Some authors suggest that it may originate from embryonic cartilaginous rests, whereas others consider it a reactive lesion related to trauma or inflammation [4,5,9]. None of these theories has been definitively proven. In addition, experimental and clinical studies on bone remodeling have highlighted the importance of mechanical loading, local biological responses, and periosteal activity in regulating bone formation [6,10,11].

Mechanical stimulation is known to induce periosteal reactions that promote osteoblast differentiation and activation, leading to localized bone formation [8-10]. In the masticatory system, complex stresses such as bending and torsion generated in the mandible by sustained contraction of the masticatory muscles, including the masseter and medial pterygoid muscles, can trigger local bone modeling responses and result in cortical thickening in areas of stress concentration [12,13].

Peripheral osteoma is considered a localized periosteal bone proliferation that typically grows slowly and behaves as a benign lesion [1-3]. In the present case, CT images clearly demonstrated a distinct interface between the lesion and the underlying normal cortex, and continuous cortical thickening suggestive of diffuse hyperostosis was not observed. Furthermore, because of the loss of molar teeth, direct occlusal loading on the left antegonial region would have been limited, making it difficult to attribute the lesion solely to occlusal stress concentration. Nonetheless, changes in occlusal relationships and compensatory alterations in muscle function secondary to molar loss may have shifted functional load to other regions and altered the local mechanical environment, indirectly contributing to tumor enlargement [12,13].

We therefore considered that mechanical stimulation in this case might have acted as an auxiliary factor in tumor initiation or growth, while other factors such as local circulatory disturbance and chronic low-grade inflammation could also have played a role. Local vascular dysregulation and microhemorrhage can stimulate the periosteum and induce new bone formation, as suggested by clinical studies on ossified subperiosteal hematoma and trauma-related ossifying lesions [14,15]. Giant mandibular osteomas associated with minor trauma have also been reported [16]. Furthermore, pathways activated under hypoxic conditions and post-traumatic periosteal responses have been reported to enhance osteoblast activity and osteogenesis, including hypoxia-inducible factor-1α (HIF-1α)-mediated regulation of periosteum-derived stem cells [17]. These mechanisms may have contributed to the development of the present lesion. However, because we did not obtain detailed histological or hemodynamic data specifically demonstrating circulatory disturbance, we cannot definitively conclude that such changes were directly involved in this case.

A genetic predisposition should also be considered, particularly Gardner syndrome, in which *APC* gene mutations are associated with multiple osteomas, intestinal polyposis, and soft tissue tumors [9,18]. In patients with multiple osteomas or gastrointestinal symptoms, careful evaluation to exclude Gardner syndrome is essential. In the present patient, clinical examination and history revealed no additional bone lesions or gastrointestinal symptoms such as abdominal pain, bleeding, or altered bowel habits, so Gardner syndrome was considered unlikely.

Radiologically, the lesion in the current patient presented as a well-circumscribed, homogeneous, radiopaque mass arising from the cortical surface of the mandible with a broad pedunculated base and without cortical destruction or periosteal reaction. These features are characteristic of peripheral osteomas of the jaws described in previous cases [3,9,19] and differ from the imaging appearances of other radiopaque lesions that must be considered in the differential diagnosis, such as exostoses, osteochondroma, osteoblastoma, osteoid osteoma, fibrous dysplasia, low-grade osteosarcoma, and ossified subperiosteal hematoma in trauma-related cases [14,15].

Exostoses are usually bilateral or multiple, develop at characteristic sites such as the palatal or buccal alveolar surface or the lingual aspect of the mandible, and tend to stabilize after completion of skeletal growth, whereas mandibular osteochondromas arise most frequently in the condyle or coronoid process and show a cartilaginous cap and continuity between the lesion and the underlying medullary bone [1,2]. In the present case, the lesion was solitary, located at the inferior border of the mandible near the antegonial notch, and showed neither a cartilaginous component nor medullary continuity on CT or histological examination, making exostosis and osteochondroma unlikely.

Osteoblastoma and osteoid osteoma typically occur in younger patients, present as mixed radiolucent-radiopaque lesions with a central nidus, and are associated with marked pain that is often disproportionate to the radiological findings [2]. Fibrous dysplasia usually exhibits a ground-glass appearance and expansion of the affected bone rather than a pedunculated exophytic mass, and low-grade osteosarcoma generally demonstrates cortical destruction, irregular periosteal reaction, and histological atypia [1,3,14,19]. In our case, the mass was uniformly radiopaque without a nidus or ground-glass change, there was no cortical destruction or aggressive periosteal reaction, and histopathology revealed only mature lamellar bone without cytological atypia, all of which support the diagnosis of compact-type peripheral osteoma rather than these entities [1,14].

Taken together, the clinical presentation and the radiological and histopathological findings indicated that the most appropriate diagnosis for this lesion was compact-type peripheral osteoma of the mandible.

Previous reviews have indicated that peripheral osteomas involving the jaw bones are uncommon and that the mandible is more frequently affected than the maxilla. They have also reported that, within the mandible, the mandibular body is the most frequent site, followed by the condyle, angle, and ramus, whereas other locations, such as the antegonial notch region, are described only rarely. These reviews also emphasize that surgical excision is the treatment of choice, that recurrence is rare, and that long-term clinical and radiographic follow-up is advisable [4,20].

The most common site was the mandibular body, followed by the condylar process, mandibular notch, and ramus, indicating that the antegonial notch is an uncommon location. Our findings are consistent with the report by Manjunatha et al., who analyzed peripheral osteoma of the mandible and found that the body was the most frequent site, followed by the condyle and angle [4].

Among the four reported cases (including the current case) of peripheral osteoma of the mandibular antegonial notch, the age at diagnosis ranged from 17 to 80 years, with a mean of 46.7 years; the present patient, at 80 years of age, was the oldest [5,7,8]. The maximum tumor diameter across these cases ranged from 23 to 50 mm, and our case had the largest lesion (Table 1). The relatively short symptomatic period prior to presentation is also noteworthy.

**Table 1:** Reported cases of osteoma arising at the mandibular antegonial notch in Japan

Year of publication	Author(s)	Sex	Age (years)	Surgical approach	Maximum diameter (mm)	Duration of symptoms (years)
1999	Watanabe et al. [7]	Female	21	intraoral	24	2
2000	Kashima et al. [5]	Male	42	extraoral	42	3
2012	Nakagawa et al. [8]	Female	17	intraoral	23	1
2024	Present case	Male	80	extraoral	50	0

To clarify the onset and growth rate, we contacted the referring dental clinic; however, the radiographs available were limited to dental periapical images of other regions, and none included the antegonial area. The patient could not recall the names of any other dental clinics he had visited in the past, making it impossible to obtain additional previous images. Considering the absence of earlier radiographic data and the patient’s tendency not to report minor symptoms, it was difficult to determine the actual time of lesion onset. Consequently, although we cannot definitively conclude that the osteoma developed within a short period, the possibility of relatively rapid enlargement cannot be ruled out.

Of the four reported antegonial peripheral osteomas, two were excised intraorally and two via an extraoral approach [5,7,8]. In our case, because of the large tumor size and the need for secure visualization and complete resection, we selected the extraoral approach. A titanium plate was used to reinforce the thinned mandibular cortex, and satisfactory functional and esthetic outcomes were obtained.

Peripheral osteomas arising at the mandibular antegonial notch, as in this case, are extremely rare and often asymptomatic in the early stages, which may delay diagnosis. When an osteoma becomes large, patients may present not only with facial asymmetry but also with functional disturbances such as malocclusion or trismus. In the present case, CT imaging clearly delineated the tumor margins and contributed significantly to diagnosis, but careful differentiation from other osseous lesions was also required. Integration of imaging findings with clinical and histopathological information is essential for accurate diagnosis.

Although recurrence after complete surgical excision of osteoma is considered rare, long-term follow-up is important to detect any recurrence at an early stage and to monitor for the development of additional bone lesions, particularly in patients with potential syndromic associations [1-3,18].

## Conclusions

We reported a rare case of a giant peripheral osteoma arising from the mandibular antegonial notch in an 80-year-old man. The lesion was successfully excised via an extraoral approach, and no recurrence was observed during 15 months of follow-up. Although the exact onset and growth rate could not be determined because of the lack of previous radiographs, relatively rapid enlargement could not be excluded. Chronic mechanical stimulation and local circulatory changes may have contributed to tumor development and growth, but their role remains speculative. This case highlights the importance of considering peripheral osteoma in the differential diagnosis of unilateral mandibular swelling, especially in atypical locations, and underscores the utility of detailed imaging evaluation and long-term postoperative surveillance.
